# Deciphering the Signal From the Noise: Caregivers’ Information Appraisal and Credibility Assessment of Cancer-Related Information Exchanged on Social Networking Sites

**DOI:** 10.1177/1073274819841609

**Published:** 2019-04-22

**Authors:** Elizabeth A. Gage-Bouchard, Susan LaValley, Julia A. Devonish

**Affiliations:** 1 Department of Cancer Prevention and Control, Roswell Park Comprehensive Cancer Center, Buffalo, NY, USA

**Keywords:** social media, Facebook, information appraisal, health literacy, information seeking, caregiving

## Abstract

With the rise in the use of the Internet for health-related purposes, social networking sites (SNSs) have become a prominent platform for cancer communication and information exchange. Studies of cancer communication on SNS have mostly focused on understanding the quantity, content, quality, and user engagement (eg, likes and comments) with cancer-related information on SNS. There is less of an understanding of when and why people coping with cancer turn to SNS for cancer-related information, and how users appraise the credibility of cancer-related information obtained on SNS. In this study, we use data from in-depth qualitative interviews with 40 primary caregivers of pediatric patients with cancer to examine how cancer caregivers engage in information appraisal and credibility assessment of cancer-related information obtained on SNS. Findings show that cancer caregivers turned to SNS for cancer-related information because information on SNS was immediate, targeted in response to specific caregiver questions and concerns, and tailored to the specific information needs of cancer caregivers. Cancer caregivers evaluated the credibility of cancer-related information obtained on SNS through assessment of the SNS user who posted the information, frequency the same information was shared, and external corroboration. Findings have important implications for cancer communication and information interventions and point to elements of SNS cancer communication that can be integrated into health professional–facilitated communication and cancer information strategies.

## Introduction

Health-related information seeking on the Internet has dramatically increased over the past decade, and over 70% of US adults report the Internet as their first source of health information.^1^ With the rise in the use of the Internet for health-related purposes, social networking sites (SNSs) have become a prominent platform for cancer communication and information exchange. The SNSs have become a ubiquitous aspect of many American’s lives,^2^ and there has been a steep increase in SNS use. In 2018, 69% of the American population reported using at least 1 social media site,^3^ and 17% communicated about health topics on SNSs.^1^ People are more likely to seek health-related information on SNS if they are coping with illness such as cancer, either as a patient or as a caregiver.^4-8^

Social networking sites have changed the nature of cancer information available on the Internet, from unidirectional passive information consumption to content that is interactively developed and expanded upon by SNS users.^2,9^ In contrast to traditional mediums for information distribution such as libraries, books, newspapers, magazines, and brochures, SNSs afford the opportunity for users to participate in a networked process of developing, sharing, and reacting to content.^2,9^ A prominent focus of cancer communication research has been to examine the type and nature of health-related communication on SNS, primarily through content analyses of social media posts, links, likes, and comments. This growing body of literature has found that, beyond being a means of accessing social support and sharing their illness-related experiences, people use SNS when seeking advice, opinions, or answers to their health-related questions.^10-17^ This type of SNS use occurs, despite patients and health-care professionals having concerns about misinformation on social media^14,17,18^ as well as the mixed results of research examining the scientific accuracy of health information provided on SNS.^19^ Another focus of cancer communication research has been to understand the quantity, content, quality, and user engagement (eg, likes and comments) in relation to information about cancer on SNS.^20-23^ The case of pediatric cancer caregivers is particularly important in understanding the role of information exchange via SNS. A pediatric cancer diagnosis requires parents to manage their child’s complicated treatment protocol, including regular clinic visits and hospital stays, all while simultaneously fulfilling other obligations related to work and family.^24^ Due to the intensive demands of caregiving, pediatric cancer parents may find themselves physically removed from traditional sources of social support (friends and family), making online platforms a potential tool for busy parents to seek information and support.^11^ While previous research has vastly expanded the understanding of the content and engagement with cancer-related information on SNS, few studies have examined when and why caregivers turn to social media for cancer-related information and how users appraise the credibility of cancer-related information obtained on SNS. This study seeks to answer these questions in order to optimally leverage SNS for cancer communication strategies and cancer information dissemination. In this article, we use data from 40 in-depth interviews with parents caring for pediatric patients with cancer to examine (1) the reasons parents choose social media as a platform for health information seeking and (2) how cancer caregivers appraise the credibility of information obtained on SNS.

### Information Appraisal and Credibility Assessment

When a person encounters cancer-related information on a SNS, they must evaluate the credibility, accuracy, and relevance of the information—a process referred to as information appraisal. Information appraisal occurs in a larger context of information discovery and subsequent use or dismissal. Dominant models of information seeking, including the Information Search Process (ISP), highlight the multiple stages information laypersons engage in as they seek, obtain, evaluate, and use information.^25^ Research has documented that Internet sources of information have increasingly changed the paradigm of information appraisal, moving away from traditional notions of need-driven information seeking, where a person identifies a need for information, finds information sources, and subsequently consumes information.^26^ In contrast, the wide-spread access to health information on the Internet, and SNS specifically, has increased information complexity and created a context, where people continually encounter information, regardless of whether or not they have explicitly identified a need for information. The ubiquity of SNS has made information acquisition (both actively sought and passively acquired) a continual process of encountering, sorting, evaluating, and acting upon, saving, or dismissing information.^26,27^ Therefore, it is critical to understand how laypersons appraise and assess the credibility of cancer-related information encountered on SNS in order to better understand how cancer information obtained through SNS shapes cancer-related attitudes, knowledge, and behavior.

The social nature of SNS adds a novel dimension to the processes through which one group of laypersons, caregivers’ of pediatric patients with cancer, appraise and assess the credibility of cancer-related information on SNS. Within the fields of information science and communication, scholars have highlighted that understanding social context is critical to understanding real-world information experiences and highlight the role of social and contextual factors on how people seek, use, and share information.^26^ This sociocultural context is critical for understanding information literacy in the era of SNS, where information seeking, appraisal, and credibility assessment are shaped within and by community engagement among SNS users.^28,29^ Central to information appraisal and credibility assessment is the perceived “cognitive authority” of information sources, which can be individuals, organizations, information platforms (eg, blogs), or institutions.^27,30^ Therefore, when assessing the credibility of information on SNS, caregivers are engaging in a process of assessing how plausible, convincing, and trustworthy the source of information is.^30-33^ Prominent models of credibility assessment highlight 4 major types of credibility: (1) presumed (trust in referring source), (2) reputed (trust due to third-party endorsement or credentials of referring source), (3) surface (presentation/design characteristics of source), and (4) experienced (previous interactions with the source).^34^ Unique features of SNS where information is shared in posts, comments, likes, and links make it important to understand how caregivers appraise, evaluate, and act upon cancer-related information obtained through SNS and assess information credibility in this new information era. This study sought to examine the reasons pediatric cancer caregivers engage with cancer-related information on SNS and how caregivers appraise and assess the credibility of cancer-related information they encounter on this platform.

## Methods

### Study Procedures

Study participants were recruited through organizations serving families coping with pediatric cancer, including an oncology hospital, pediatric cancer-related organizations, and social media groups related to pediatric cancer. For in-person study recruitment, the pediatric psychologist approached potential participants, described the study, and asked parents’ permission to have their contact information shared with the researchers. Study advertisements were also posted on pediatric cancer-related social media groups and asked interested parents to contact the study team by phone or e-mail. Inclusion criteria included being a parent of a child on active therapy for acute lymphoblastic leukemia and verbal English fluency. In the case of 2-parent families, the parent with primary caregiving responsibilities was asked to participate in the study. The study was overseen by the University at Buffalo Institutional Review Board, and all participants provided verbal informed consent. Interviews lasted between 30 minutes and 1 hour. All data were collected between June 2015 and August 2016.

The research team created a semi-structured interview guide to drive the conversation toward topics related to the experiences of cancer caregiving. The interview guide can be found in Appendix A. The interview guide included broad prompts aimed to capture different aspects of cancer caregiving (eg, daily caregiving tasks, administering medication, assessing pain, and accessing cancer information), while allowing participants' narratives of their own experiences to lead the conversation. Interviewers were trained to use follow-up prompts to capture additional information introduced by the participants. Two interviewers (the first and second author) conducted all interviews. Both interviewers have extensive experience in qualitative data collection and met weekly during data collection to review interview data as it was being collected. The principal investigator for this study (first author) reviewed all data files as interviews were being collected to ensure interview fidelity. During the first 7 interviews, use of SNS for cancer-related information was a common theme emerging from interview data. Due to this, during weekly interviewer meetings, we decided to add several additional questions to the interview guide specifically related to experiences using SNS for cancer-related communication (questions 11-14, see interview guide in Appendix A). All interviews were audio-recoded and transcribed verbatim. Participants received a US$50 gift card upon completion of the interview.

### Sample Characteristics

Our sample included 39 mothers and 1 father. Thirty-seven participants were white, 2 were African American, and 1 was Hispanic. Thirty-six participants were married and 4 were not married. Four participants had a high school degree or less, 9 had an associate’s degree or some college, 14 had a bachelor’s degree, 12 had a graduate degree, and 1 did not report educational attainment. Thirteen participants had a total household income greater than US$100 000 annually, 16 were between US$50 000 and US$99 999, 6 were between US$35 000 and US$49,999, and 5 had a total household income less than US$34 999 annually. Twenty-three participants worked full time, 4 worked part time, and 13 did not work for pay. Child’s age at cancer diagnosis ranged from 1 to 18 years old, and 6.5 years old was the mean age at diagnosis. Child’s time since diagnosis ranged from 5.8 months to 57.9 months, with a mean time since diagnosis of 21.2 months (see Table 1).

**Table 1.** Sample Characteristics.

	n (%)
Relationship with the patient	
Mother	39 (97.5)
Father	1 (2.5)
Race/Ethnicity	
Black	2 (5)
White	37 (92.5)
Hispanic	1 (2.5)
Employment status	
Part time	4 (10)
Full time	23 (57.5)
Not working	12 (30)
Unknown	1 (2.5)
Marital status	
Married	36 (90)
Not Married	4 (10)
Education	
High-school diploma or less	4 (10)
Some college	9 (22.5)
Bachelor’s Ddgree	14 (35)
Graduate degree	12 (30)
Not reported	1 (2.5)
Household income	
US$34 999 or less	5 (12.5)
US$35 000-US$49 999	6 (15)
US$50 000-US$99 999	16 (40)
US$100 000 or more	13 (32.5)

### Data Analysis

Three members of the research team, which included both interviewers and a naive coder who had not been involved in data collection, read each data file and wrote coding memos that highlighted key ideas, codes, and themes that the team member identified in an initial reading of the data. Each team member prepared coding memos independently to allow sharing of initial impressions of the data prior to group discussion and input. The data analysis began with a meeting of the research team to discuss impressions from field experiences and prominent themes in the data files. An initial list of codes was developed based upon the interview guide, major research questions, and a review of the literature. Three members of the research team then coded each data file and any coding discrepancies were discussed and resolved at team meetings. Throughout data analysis, each team member wrote memos that reflected on the codes, patterns among the codes, and early ideas about themes in the data. At peer debriefing meetings, team members reviewed and discussed points identified in coding memos.^35^ The team used a multiple coder strategy to increase the reliability of data analysis, and 3 team members each independently analyzed the data.^35^ Data analysis was guided by a thematic analysis approach to categorize codes and identify themes.^36^ The themes were identified based upon frequency of codes, code patterns, and the context and meaning of codes for groups of respondents.^36^ Through an iterative process, themes were discussed and refined at team meetings.

## Results

Caregivers identified aspects of information exchange on SNS that made it an appealing and helpful tool during the cancer experience such as information immediacy, relevance, and composition of knowledge peers (other cancer parents). Caregivers engaged in information appraisal strategies that relied on group consensus, source evaluation, and corroboration with health-care professionals.

### Why Cancer Caregivers Use SNS: Immediate, Targeted, and Tailored Information

When describing their information-seeking practices, cancer caregivers commonly reflected on the appeal of the immediate nature of obtaining information through social media. Due to the interactive nature of SNS, parents can post questions and receive rapid answers that are tailored to their specific information needs. For example, one mother described:

The kinds of things I’ve looked for on [Facebook] was when my son was entering a new phase of treatment, what was that phase like for other people…Today I put something on [Facebook] about, he’s been really nauseous and throwing up, and I put something on [Facebook] about dosing…Within six minutes of posting it, people had responded and said that their kid who was the same weight as my kid was on the same dose so it must not be that issue…It’s fast information from not a medical source, but I would say that sometimes cancer parents are more knowledgeable than doctors about certain things. It saved me having to email the doctor, that probably would have been my next step, now that four people said that they kid is the same size and on the same dose, I don’t think it’s worth my time to email the doctor and check that out. (R18)

As Respondent 18 described, Facebook was an appealing source of cancer-related information because information acquisition was fast and multiple parents responded similarly, suggesting that their answers were credible. As her experience illustrates, information exchange on SNS was also tailored to specific questions and experiences.

Caregivers also reflected on the connections to other cancer caregivers through SNS, and these connections among knowledgable peers enhanced the appeal of SNS as a source of cancer-related information. Respondent 15 explained,

It’s people who have been through the experience so they’re not messing around. They tell it like it is and they’re just blunt and honest about it. (R15)

The SNSs afford the opportunity to form communities of patients or caregivers experiencing the same cancer diagnosis, and these communities provide the opportunity to share tailored information informed from having shared experience with cancer. As respondent 18 described, she perceived other cancer caregivers as sometimes being as knowledgeable about cancer caregiving as physicians. In addition to the perceived expertise of other cancer caregivers, respondent 15 highlights that due to their shared experience, peers can share information in a “blunt” and “honest” way, which was an appealing aspect of turning to SNS for cancer-related information.

Finally, rather than sifting through books or articles on their own, SNS allowed caregivers to post specific questions and receive direct and tailored responses. A common theme in parents’ posts centered on soliciting tips for helping children take their medication. For example, respondent 10 described:

I didn’t learn about this magic pill cup until I posted a question ‘how do you get your child to take pills easier?’ Because swallowing pills is not something that [patient] has ever had to do prior to diagnosis and it was a struggle…So I found this oral pill cup and ordered it and said okay, this has been a godsend and it’s because I asked the question on the [Facebook] page. (R10)

As respondent 10 explained, SNS afforded the opportunity to ask targeted questions and receive tailored information directly relevant to their information needs.

### A Networked Process of Trust Building

When discussing how they evaluated information obtained through SNS, many parents reflected on the characteristics of the person who posted the information. Within this theme, some parents ascribed more credibility to posts by other parents of pediatric patients with cancer. For example, respondent 13 explained:

I’ve grown to trust most [cancer] moms [that post on social media]. They’re very understanding. They’ve been through it longer than I have. I’ve been going through this with her for almost two years now, it’s hard. And they’ve been through it for 7 years total. (R13)

As respondent 13 described, some parents felt that information from other cancer caregivers was credible due to shared experience, accrued expertise, and understanding of pediatric cancer care. Parents also relied on past experience with specific social media users to evaluate the credibility of information shared. Respondent 30 explained:

When I asked the question, the one woman who responded, I know she’s somebody who consistently responded. Very educated on the matter. (R30)

As respondent 30 explained, previous experience with specific social media users shaped caregivers’ perceptions of the level of credibility in the information being shared depending upon caregivers perception of how “educated on the matter” the person posting information is as well as how frequently or consistently another parent was perceived to respond. Similarly, caregivers relied on the nature of previous posts when evaluating the credibility of information shared. Respondent 10 described:

There are some that I see and they’re all negativity. I don’t want to be reading all negative stuff. I want a mix of things, I want some bad, I want some good. I want to hear the pros and the cons. A good balance…There’s always going to be those moms that are doomsday all the time. It’s always always bad and you know, I’m just gonna skip over that person, I’m not gonna read it because it’s all negative. I don’t need all that negativity, that’s not what I’m looking for. (R10)

Previous experience with social media users shaped caregivers’ perceptions of the dispositions of each social media user. Caregivers also used social media as a way to crowdsource information and used the number of users expressing similar information as a way to evaluate the credibility of information being shared. Respondent 12 described:

You can have ten other people saying the same things…You kind of using it as your sounding board…like can you talk me off the wire? (R12)

Caregivers used social media as a way to quickly receive information from multiple other users who acted as a “sounding board.” Moreover, caregivers noted the supportive nature of having a group validate their experience. When multiple users shared the same information, caregivers often ascribed more credibility to the information being shared.

### Information Appraisal Strategies: Source Evaluation, External Corroboration, and Physician Consultation

Similar to traditional dimensions of credibility assessment, caregivers also examined aspects of the post or link to evaluate the credibility of information shared on SNS. Some caregivers evaluated the source of the information beyond characteristics of the SNS user posting the information. For example, Respondent 32 described:

I’ll look at the link and if it looks like it’s coming from somewhere reputable or a place that I can trust, I just kind of use my intuition with that and just make sure that the information is relevant and tested. (R32)

Caregivers looked at the original information source through evaluating the link and institutional credibility of sources of information. Similarly, respondent 34 explained:

Most of them are research articles. I’m not one to research but I look at the links.

As respondents 32 and 34 explained, information that linked back to trusted institutional sources was perceived to be credible. In addition to posts that contained links to information sources, some caregivers described conducting additional research to evaluate the credibility of information obtained on social media. Respondent 26 described:

There’s a lot of sharing information and somehow I had come across a mother’s post and she was discussing this study so that’s where I then Googled it and found the link to the study. Then after that I immediately called [my child’s] doctors to discuss it. (R26)

Finally, as described by respondent 26, many caregivers described discussing information obtained on SNS with their child’s doctor. For example, respondent 38 explained, “I ask the doctors. I always go through the doctors no matter what I hear.” Caregivers described using SNS as a source of information, but ultimately using information obtained on SNS as a way to inform and guide further conversations with their child’s oncologist. Respondent 30 explained:

It wasn’t, I’m gonna go by what she said, it was, here’s another piece of the puzzle. Another thing for me to consider and to go back and talk to my oncologist…It wasn’t like, I’m gonna start doing this, it was more, these are question I need to take back to the oncologist and talk about with her. (R30)

Caregivers described soliciting and obtaining information on SNS that equipped them to ask informed and targeted questions of health-care providers. Caregivers also highlighted the need for different methods of credibility assessment based on the type of information being obtained. For example, caregivers described vetting medically oriented information with physicians and relying on SNS for more caregiving-related information. For example, respondent 19 explained:

I don’t always let myself believe that everything is true. I think everyone has their own story and everything affects everybody differently. What I do is try to gather as much information from other people, and then I go back with the oncologist or with the Internet, and sort out with myself what is happening with us. Do I trust another mom telling me ‘he’s got appendicitis’? Absolutely not. But maybe ‘giving him a banana might help his tummy’ or something like that. If anything major needs to happen, it has nothing to do with anybody but the doctors or nurses. (R19)

As respondent 19 described, SNSs were a source of caregiving information about managing their child’s symptoms at home. In contrast to information that allowed parents to self-manage, parents identified that some types of medical information, such as diagnosing symptoms, should be discussed with physicians.

## Discussion

The increase in cancer communication on SNS has created the need to explore how users evaluate the information on these platforms. Recent research has also documented that people experiencing cancer use SNS as a source of cancer-related communication and information. The rise in cancer-related communication on SNS has led to a need to understand how cancer caregivers are engaging with this new platform for health communication. Within this, 2 major lines of research have emerged. First, research has examined the types and nature of health-related communication on SNS.^10-17,19^ Second, research has examined audience engagement with cancer-related information posts on SNS by examining what types of posts receive the most comments, shares, likes, and emoji reactions.^6-8,20-23^ This previous research has answered critical questions surrounding the types of cancer-related information available on SNS and differential amplifications of certain kinds of cancer-related information on SNS by way of audience engagement. However, little is known about how SNS users appraise and assess the credibility of information they obtain on SNS. This is a critical gap, as SNS users subsequently use these information appraisals when determining whether or not they dismiss or act upon the information obtained on SNS.

In this study, we used data from 40 in-depth interviews with parents caring for pediatric patients with cancer to examine factors influencing caregivers’ choice to use social media for cancer-related information and how cancer caregivers appraise the credibility of information obtained on SNS. We found that cancer caregivers turned to SNS for cancer-related information, because information on SNS was immediate, targeted in response to specific caregiver questions and concerns, and tailored to the specific information needs of cancer caregivers. These findings add to the understanding of why people coping with illness use SNS as an information source. Amid the many demands of being a cancer caregiver, participants in our study described SNS as an appealing information source because SNS offered a mechanism to receive answers to health-related questions at any time of the day without delay. The SNS also afforded caregivers the opportunity to receive timely information in response to specific questions as they arose, foregoing the need to access information using traditional sources (ie, books and articles), sift through a large volume of information, and determine what was relevant to their particular question. Due to the composition of the study sample (predominantly white, educated, and female caregivers), findings from this study may not be applicable to all pediatric cancer parents. Similarly, our sample included caregivers of pediatric patients with acute lymphoblastic leukemia. Caregiving context can shape caregiving tasks, caregiving burden, and information needs, and caregivers of patients with different illnesses may subsequently use SNS differently. An important direction for future research is to examine differences and similarities in SNS across caregivers in different illness contexts.

Our findings also show that cancer caregivers evaluated the credibility of cancer-related information obtained on SNS through an assessment of the SNS user who posted the information, how frequently the information was shared, and external corroborations of the information. Prominent models of information appraisal highlight the importance of the cognitive authority of information sources as critical in assessing credibility of information.^30,34^ Our findings extend these conceptual models and highlight peer experience as an important factor that enhanced perceived credibility of information exchanged on SNS. Our findings also highlight the importance of integrating social contextual dimensions into understanding of credibility assessment process on SNS. Because SNSs are based upon a networked process of information exchange, context-dependent social strategies were an integral component of how cancer caregivers vetted cancer-related information obtained on SNS. Cancer caregivers deemed information as more credible if it was offered by an SNS user who was also a cancer caregiver and based upon the assessment of the SNS user’s knowledge and perspectives in previous SNS interactions.

## Implications

These findings point to important directions for interventions and future research. Caregivers in our study relied on SNS as a source of cancer-related information because SNS connected them to knowledgeable peers as well as immediate and reactive information relating to their specific questions or concerns. These components could be integrated into health professional–facilitated communication and cancer information dissemination strategies. For example, since this study’s findings highlight that caregivers find useful information on SNS to supplement their informational and social support needs during the caregiving experience, health-care providers might direct cancer caregivers to consult SNS communities for day-to-day information about dealing with nuanced aspects of cancer caregiving (eg, tips for swallowing pills and sharing helpful stories.). Moreover, since findings from this study reveal that the levels of trust caregivers have in their peers with shared experience can parallel the levels of trust they have in health-care providers, caregiver interventions should harness the power of peer-to-peer information exchanges and specialized guidance as opposed to traditional, top-down authoritative disseminations of information from health-care professionals. Application of this particular finding might take the form of caregiver-led efforts to create specialized informational guides for their peers on topics such as managing day-to-day challenges of the pediatric cancer caregiving experience.

Finally, a prominent theme among cancer caregivers in our sample was externally corroborating cancer-related information obtained on SNS with their child’s oncologist. One implication of these findings for clinical practice is that oncologists should be aware that conventional notions of health information authority and credibility are shifting. Our data support this multifaceted approach to credibility exists (ie, cancer caregivers use several indicators for reliability, not just deferring to a physician). This requires a shift in how oncologists approach patient–provider communication in this new era of increasing patient autonomy.^37^ Oncologists should initiate discussions to find out whether cancer parents and caregivers are engaging in exchanges of cancer-related information on SNS so that oncological team can create a space to give parents opportunities to corroborate what they’re reading on SNS.^37^ Our findings show that cancer caregivers used number of similar responses as a mechanism to assess the credibility of information obtained on SNS. While this may be effective if the information being shared is medically and scientifically correct, it is cause for concern if misinformation is being widely shared and amplified.^18^ These findings highlight the need for clinicians to acknowledge the use of SNS as a source of cancer-related information and proactively offer patients and caregivers opportunities to discuss information obtained through SNS within the clinical encounter.

## Appendix A

### Interview Guide

1. First, can you think back to yesterday and walk me through your day.
2. When you have to give your child oral medication what exactly is your routine? (do you watch them take the pills?)
3. What about pain medication, what has your strategy been to determine when and how much pain medication your child needs?
4. There is a lot to balance with figuring out insurance, caring for your child, and fitting appointments into your family life. How did you manage that?
5. Was it ever difficult to follow the doctor’s instructions?
6. How did you get information about cancer and caring for your child?
7. How did you learn how to do the more technical aspects of caregiving, like giving your child medications, managing their port, etc?
8. Do you feel that you received enough information about providing care for your child?
9. Have you ever needed to ask to modify the medications (due to side effects, or difficulties administering?) What did you do?
10. Have you connected with other parents of cancer patients?
  - [If yes] What kinds of things did you talk about?
  - [If yes] Was this helpful?
11. Do you use any social media sites, for example Facebook, Twitter, or Instagram?
  - [If yes] have you used [Facebook, Twitter, etc] in any ways related to your child’s cancer?
  - [If yes] Have you obtained information related to cancer through [Facebook, Twitter, etc]?
  - [If yes] What kind of information?
  - [If yes] Was it helpful?
  - [If yes] How did you decide if it was reliable?
12. Have you used [Facebook, Twitter, etc] to ask people for help with daily tasks such as help with meals or household chores?
  - [If yes] What kind of responses did you get from your [Facebook friends] to these requests?
  - [If yes] Is that how you hoped they would respond?
13. What about emotional support, do you find [Facebook, Twitter, etc] was a source of emotional throughout your child’s care? How so?
14. Have you used [Facebook, Twitter, etc] to help advertise fundraisers related to your child’s cancer?
  - [If yes] What kind of responses did you get from your [Facebook friends] to these requests?
  - [If yes] Is that how you hoped they would respond?
